# The current situation in education and training of health-care professionals across Africa to optimise the delivery of palliative care for cancer patients

**DOI:** 10.3332/ecancer.2014.492

**Published:** 2014-12-11

**Authors:** FM Rawlinson, L Gwyther, F Kiyange, E Luyirika, M Meiring, J Downing

**Affiliations:** 1 Department of Palliative Medicine, Cardiff University, Velindre Hospital, Cardiff CF14 2TL, Wales, UK; 2 Hospice Palliative Care Association of South Africa and Senior Lecturer, palliative medicine, University of Cape Town Anzio Road, Observatory, Cape Town, South Africa; 3 African Palliative Care Association, PO Box 72518, Plot 95, Dr Gibbons Road, Makindye, Kampala, Uganda; 4 Sarah Fox Convalescent Hospital and PATCH-SA (SA Children’s Palliative Care Network), Lecturer, palliative medicine, University of Cape Town Anzio Road, Observatory, Cape Town, South Africa; 5 Honorary Professor, Makerere University, PO Box 7062, Kampala, Uganda; International Palliative Care Consultant

**Keywords:** palliative care, education, e-learning, policy, professional development, outcome measurement, Africa

## Abstract

The need for palliative care education remains vital to contribute to the quality of life of patients, both adults and children, with cancer in Africa. The number of patients with cancer continues to rise, and with them the burden of palliative care needs. Palliative care has been present in Africa for nearly four decades, and a number of services are developing in response to the HIV/AIDS epidemic. However, the needs of cancer patients remain a challenge. Education and training initiatives have developed throughout this time, using a combination of educational methods, including, more recently, e-learning initiatives.

The role of international and national organisations in supporting education has been pivotal in developing models of education and training that are robust, sustainable, and affordable. Developing a material for education and professional development needs to continue in close collaboration with that already in production in order to optimise available resources. Seeking ways to evaluate programmes in terms of their impact on patient care remains an important part of programme delivery. This article reviews the current situation.

## Introduction

The burden of cancer in Africa continues to increase. Survival rates are still affected by late detection, delay in seeking diagnosis, the lack of availability of technical support for diagnosis, lack of resource for treatment and the availability of palliative care [1, 2]. In 2005, the World Health Organisation (WHO) estimation of the need for palliative care (as 1% of a country’s total population) was approximately 9.67 million people across Africa. Although that figure includes all conditions, cancer remains a significant proportion [3, 4]. In 2012, there were an estimated 14.1 million new cancer cases and 8.2 million cancer-related deaths globally. More than half (57%) of the new cancer cases and nearly two thirds of related deaths (65%) occurred in less developed regions of the world [5]. The number of new cancer cases in Africa is predicted to increase to 1.28 million, with 970,000 deaths each year by 2030 [6].

## Palliative care and hospice care—definitions

The WHO summarises palliative care as an approach that improves the quality of life of patients and their families facing the problems associated with life-threatening illness, through the prevention and relief of suffering by means of early identification and impeccable assessment and treatment of pain and other problems, physical, psychosocial, and spiritual [7]. From the origins of palliative care at St Christopher’s hospice in London, UK, in 1967, palliative care services have developed with the term ‘hospice care’ being variably used to describe the care they provide. However, globally, there is a lack of consensus among providers: some using the term ‘hospice’ to describe the philosophy of the team and others an actual physical location. There is still variability in what a service describing itself as ‘hospice’ provides [8]. The Worldwide Hospice and Palliative Care Alliance (WHPCA) describes hospice services as ‘usually community-based services that deliver palliative and end of life care in all settings for patients, families and carers’ [9]. The common issue for both palliative care and hospice care is the comprehensive interdisciplinary team providing that care.

## Impact

The impact of palliative care on patients’ quality of life and survival is well documented [10]. Increasing the availability of palliative care provision for patients with cancer is an urgent need, in addition to increasing efforts to improve early detection and access to treatment [11–15]. Increasing palliative care education and training support to underpin this provision is vital.

Since Island Hospice in Zimbabwe was established in 1979 as the first palliative care service in Africa, there have been major developments throughout the continent; however, expansion and developmental needs continue [16–18]. The Cape Town Declaration of 2002 asserted palliative care and pain relief as a human right and the need for palliative care to be incorporated into national health-care strategies. The declaration further highlights the need to train all the members of health-care teams and providers in palliative care at all levels of education [15]. The African Palliative Care Association (APCA), formed in 2004, continues to be the leading organisation for Africa on advocacy and policy development and provides support for training and delivering services across the continent.

## Challenges for the provision of palliative care in Africa

However, despite these strengthening foundations, there are still a number of challenges. A survey of hospice and palliative care services on the continent showed 45% of African countries with no identified hospice or palliative care activity and only 9% could be classified as having services approaching some measure of integration with mainstream health provision [19]. The continent continues to face a shortage of health-care professionals: As an example, per 100,000 people, Tanzania has two doctors and 37 nurses; Mozambique, three doctors, 21 nurses; and Cote D’Ivoire, 12 doctors, 60 nurses. South Africa has only one specialist nurse per 39,400 cancer patients [20]. Access to palliative care services, especially in rural areas, could be greatly increased if many health-care professionals are trained and permitted to deliver aspects of holistic care, including prescribing of morphine. Uganda has changed existing legislation to allow some doctors’ roles to be ‘shifted’ to specially trained nurses, for example, enabling them to prescribe oral morphine to patients with moderate to severe pain [21]. As a model of how to increase palliative care provision for patients including those with cancer, this is being considered by other countries.

Inevitably, provision varies according to context, but Africa, in addition to shortages of doctors and nurses with adequate skills and training in palliative care, currently faces extremely restricted access to the multi-disciplinary teams needed to deliver holistic palliative care. Most health services have no palliative care team, with health-care providers working with different colleagues at different times. Non-Governmental Organisations (NGOs), hospices, (the term ‘hospice’ relating to the organisation rather than a physical location), and faith and community-based organisations provide some support in some areas. The reality is often simply a nurse and a community volunteer, working with family. Africa’s low urbanisation means services often need to be provided to low-density populations across vast rural areas (Namibia averages two people per km^2^; Sudan’s population is 80% rural or nomadic). Many health professionals prefer to work in urban areas, so rural health provision is often left to community- and home-based volunteers, able to deliver care that is only supportive in nature [20].

Opioid prescribing for the effective management of cancer pain continues to be an issue and a challenge for health-care professionals. There is evidence of continued opioid phobia amongst medical colleagues though lack of understanding and lack of training [20] and continued stringent regulations regarding supply and availability [22].

Using the integration of palliative care into national health policy as a measure of development of palliative care services, to date only three African countries have palliative care integrated into their national health policies and strategies (Uganda, South Africa, and Tanzania). Swaziland, Rwanda, and Zambia have developed draft policies that are subject to approval by their health ministries. Five countries across Africa have palliative care integrated in the curriculum of health professionals, and two (Uganda and South Africa) have recognised palliative care as an examinable subject. Funding and resources for cancer and cancer treatment remain a low priority for some countries. The data from the International Atomic Energy Agency estimated that 16 out of 53 African countries have no radiation treatment centres, with 13 countries where availability is unknown [2].

## The provision of palliative care education for cancer across Africa

### Barriers to education in palliative care

Education is a key and vital contribution to the effectiveness, recruitment, retention, and sustainability of all palliative care teams [23]. Palliative care teams in their turn have an important role to play in helping to support and educate colleagues. There are a number of resource challenges when considering delivering education across Africa: availability of educators, the impact on organisations of time away for learners for face-to-face sessions, travelling distances, logistics and cost [24–26]. These challenges are compounded by other factors, such as how best to teach values, beliefs, and ethics, the numbers requiring training, a lack of recognition of palliative care (and in particular of palliative care for children), and the provision of mentorship and supervision [27, 28].

WHO recommended in 2004 that governments include palliative care in training curricula for health workers at all levels [29] and such education programmes for palliative care have been developing across the region over the past 20 years, with a variety of courses available for health and social care professionals, volunteers, religious leaders, teachers, and others involved in the provision of palliative care services.

### Providers of palliative care education in Africa

- National Associations, e.g., Kenya Hospices and Palliative Care Association (KEHPCA), Hospice and Palliative Care Association Of South Africa (HPCA), Palliative Care Association of Uganda (PCAU), Palliative Care Association of Malawi (PACAM)
- Specific palliative care organisations, e.g., Island Hospice, Hospice Africa Uganda, Mildmay, Nairobi Hospice
- Universities, e.g., University of Cape Town, Makerere University, Kenya Medical Training College, and the International Technological University of Tanzania
- The African Palliative Care Association (APCA)
- Additional global organisations: International Association for Hospice and Palliative Care (IAHPC), Worldwide Hospice and Palliative Care Alliance (WHPCA), Virtual University for Cancer Control net (VUCCnet).

## The role of palliative care education providers

### National palliative care associations

National palliative care associations contribute to both education and mentorship. Experience has shown that practitioners are at risk of losing their newly acquired knowledge and skills if they return to the workplace without support to implement their learning. In South Africa, the transference of skills to the workplace is a focus of the HPCA’s training courses. Learners have an initial training period and then are required to use their knowledge and skills in their work and document this activity as the assessment of their learning. They then return to the classroom, real or virtual, to consolidate their learning.

The National Palliative Care Associations also have a role in developing or adapting curricula, facilitating accreditation with national accrediting bodies, presenting training, assessment of learning and moderation of courses. They may also influence professional bodies to pronounce on the requirement for palliative care amongst health-care professionals; and may influence and support tertiary education institutions to integrate palliative care training into basic, intermediate and specialist training courses.

### Community volunteers

The role of community volunteers in palliative care in Africa cannot be underestimated [30], and training programmes have been provided specifically aimed at this group. For example, in Namibia, training was rolled out to community volunteers with Catholic Aids Action (CAA); in Zimbabwe, community volunteer training has been provided through Island Hospice and HOSPAZ; in South Africa HPCA has developed similar programmes.

### Universities and other health-care training facilities

- The universities play a role in integrating palliative care into undergraduate and postgraduate education. Palliative care has been included in the undergraduate curriculum in Uganda and South Africa for many years and strategies for implementation into curricula are currently in development in Namibia, Botswana, Malawi, Tanzania and Kenya, with each country at a different level of integration.
- The Diana Princess of Wales Memorial Fund provided guidance and funding to implement the integration of palliative care training into medical and nursing schools in Kenya, Malawi, Tanzania, and South Africa.
- In 2001, the University of Cape Town, with support from the University of Wales College of Medicine, now Cardiff University, developed the first postgraduate palliative care diploma and degree (M.Phil.) in the region. The programmes, accessible to all health professionals, have a focus on both adult and children’s palliative care [31]. The Institute of Hospice and Palliative Care in Africa (IHPCA) at Hospice Africa Uganda, developed a 9-month course for prescribers, completion of which enabled nurses and clinical officers to be recognised as prescribers. Whilst initially a certificate level course has since been upgraded to a diploma programme, the work is ongoing as to how this can be expanded to other countries.
  Other postgraduate courses have also been established: Nairobi Hospice in affiliation with Oxford Brooks University (UK), Mildmay International in affiliation with the University of Manchester (UK), Hospice Africa Uganda in affiliation with Makerere University, and more recently the International Medical Technological University in Tanzania, Kenya Medical Training College, and Mildmay Uganda who have commenced a diploma in children’s palliative care with a curriculum initially funded by The Diana Princess of Wales Memorial Fund. Other Universities in the region are looking at developing palliative care programmes, for example, the Institute of Health Sciences in Gaborone, the International University of Management in Namibia, and the College of Medicine and Health Sciences at the University of Rwanda.
- Certificate courses in palliative care have been run for many years across the region. For example, Uganda, South Africa, Kenya, Zambia, Swaziland, and Botswana amongst others. HPCA in South Africa has developed a series of locally accredited programmes which have been rolled out throughout the country.

### The African Palliative Care Association (APCA)

APCA, working with Ministries of Health, National Palliative Care Associations, palliative care donors and palliative care implementers, has worked to provide training and education opportunities through facilitating the following approaches within countries and across borders. In particular APCA has facilitated:

- The development of a core curriculum for introductory palliative care training of health-care professionals [24] and core competencies [25]
- Educational and awareness promoting visits for policy makers
- Clinical placements for palliative care providers
- Supporting multi-disciplinary palliative care providers to attend certificate, diploma and degree courses
- Supporting the integration of palliative care into curricula of nurses, doctors, and social worker
- Research training for palliative care professionals through the African palliative care research network

APCA has also supported the development of key documents to support palliative care education in Africa (see Table 1).

One of APCA’s critical roles continues to be advocacy for palliative care integration in existing health-care education and training programmes. In collaboration with its membership and partners within and outside Africa, APCA continues to provide technical assistance and resources to African Governments, national palliative care association, oncology centres, and other key stakeholders to achieve this goal [37].

Working with partners in Europe and the United States, APCA has also supported palliative care scholarships for medical, nursing, and social work professions to study palliative care. To date, more than 70 professionals from 17 countries have received these scholarships. In addition, APCA works with regional and international bodies such as the WHO and the African Union to strengthen regional frameworks for palliative care, including the availability and accessibility to essential medicines. Training has focused not only on clinical palliative care issues for both adults and children, but also related issues such as ‘training of trainers’, palliative care research, management, legal issues, and leadership training.

Provision of training is often limited due to funding, which has led to various grants and projects already underway in the region which have a focus on training. A recent example is the THET Multi-country partnership project on ‘*Strengthening and integrating palliative care into national health systems through a public health primary care approach in 5 African countries to contribute to meeting the targets of Millennium Development Goal 6 (MDG 6)*’ commenced in 2012 through a partnership of the University of Edinburgh, APCA, and the Makerere Palliative Care Unit (MPCU). This project has enabled training in palliative care, training of trainers, community sensitisation, research and children’s palliative care to be provided in Kenya, Uganda, Zambia, and Rwanda, and such training is a key component of the integration of palliative care into the national health system.

### Global organisations

- Working alongside both national and regional organisations, international organisations such as International Children’s Palliative Care Network (ICPCN), International Association For Hospice And Palliative Care (IAHPC), Worldwide Hospice Palliative Care Alliance (WHPCA), and the World Health Organisation (WHO) all have an important role to play in capacity building for education within the region. As the international leader for children’s palliative care, ICPCN has provided training programmes on children’s palliative care across the region including in South Africa, Uganda, Namibia, Rwanda, Kenya, Tanzania, Swaziland, Zambia, and Sudan, amongst others, alongside their e-learning programme. ICPCN also has a role in advocating for children’s palliative care and its integration into national health structures.
- IAHPC provides access for members to many online databases and journals that people within the region would otherwise not have access to, thus providing an essential service for those undertaking training within the region. In addition, their bursary scheme has supported over 120 participants from across the region to attend training programmes including diploma, degree, and masters programmes.
- WHPCA produced the ‘Palliative Care Toolkit: Improving care from the roots up in resource-limited settings’ [38] which has been translated into eight languages and the companion ‘Training Manual for the Palliative Care Toolkit’ [39]. Hospice UK, provides the secretariat for the WHPCA and has sourced funding from the Wolfson Foundation to support international bursaries for palliative care. The total number of grants awarded worldwide were 461 and to Africa 250.
- Virtual University For Cancer Control (VUCCnet) has collaborated with partners in Bristol (UK) and Cardiff (UK) to produce an open access online palliative care education programme that was launched in 2014 [40]. To date there have been 256 learners who have completed modules, and all the modules have been accessed, the most often accessed being the basic principles of palliative care (14%) [41].

## Modes of delivering education for palliative care in cancer

### Online learning

In order to increase accessibility and availability of low-cost education in palliative care across the region, various modes of delivery have been utilised, and whilst the majority of training in the past has been done face-to-face, other options have developed, such as distance learning and, more recently, e-learning. Using technology to deliver education is not a new concept. Increasing internet availability and access to mobile phone technology provides the opportunity to deliver education on a far greater scale [42–44].

Delivering education ‘online’ in isolation has been challenged as an effective mode of delivery, but for pragmatic reasons, providing the educational content and framework is sound, it may deliver the greatest potential for learning at low cost across the widest number of participants [42–43]. Massive online learning communities (MOOCs) are in their early stages but few exist specifically for palliative care and none within Africa. Combining methodology, such as using e-learning and a local placement facilitates embedding learning within a practical setting.

Examples of e-learning palliative care in cancer courses include:

- VUCCnet/*e*cancer open access education modules which offer information on 20 topics relevant to palliative care in the African setting and provide health-care professionals with information, interactive tasks and suggested resources. APCA’s E-learning guide on pain management which is based on ‘*Beating Pain: A Pocket Guide for Pain Management in Africa*’ [32] offers opportunity for Africa’s health-care professionals to learn about effective pain management using adult and children specific case studies. Currently, APCAs in collaboration with the ‘Treat the Pain’ Programme of the American Cancer Society, are also implementing a project in Uganda to determine if e-learning is as effective as classroom training for pain relief. The project is also exploring models for using e-learning to rapidly scale up clinical training for first-line prescribers of morphine by increasing access to training and reducing the cost of training. The project has a research component, and the results are expected to add to the limited evidence available that compares e-learning to classroom learning.
- The International Children’s Palliative Care Network (ICPCN) has developed online training in children’s palliative care [45]. Courses are endorsed by the University of South Wales, and are free of charge to participants. Initial courses focus on understanding the concept of children’s palliative care, pain assessment and management, psychological issues and communicating with children, play, grief and bereavement, and death and dying. Courses are available in a variety of languages to increase accessibility in the region, e.g., English, French, Portuguese and Spanish, and further courses are under development. Since the ICPCN programme was introduced, over 800 participants have accessed the courses, from 76 countries, 15% of whom have been from Africa.
- The University of Cape Town postgraduate courses are presented as mixed mode programmes using a blended approach to learning. There are two residential weeks in each year, and the bulk of the content is presented in the e-learning format using a Vula, an e-learning platform powered by Sakai as the mode of delivery. Online reading materials and online discussion forums are the key learning methods for these courses. There are opportunities for more formal links with local organisations to be built in terms of observing practice and inter-professional support. The e-platform can be used to deliver the course content, patient, and case studies as well as training scenarios. In addition, the assessment of students and their lecturers can be done online. Face-to-face skills and physical mentorship can then be achieved through a clinical placement at a site with capacity to provide the support for the identified skills.

## The impact of palliative care education

Evaluating education programmes may inform providers of the quality of that training, and postulated changes in learner behaviour as a result of the training but there continue to be challenges in rigorously evaluating the impact of the training on patient care [46–48]. Several evaluation studies have been completed looking at some longer courses on palliative care, for example, the impact of a palliative care modular training programme in rural Uganda [49] and studies looking at the impact of programmes in South Africa [50].

An evaluation carried out to look at ICPCN’s e-learning programmes, alongside some taught training they have conducted in the region, showed that 80% of participants undertaking the e-learning programme and 90% of those studying by the face-to-face training, found it ‘very useful’ for their practice, with the difference being attributed to the fact that the face-to-face trainings were tailored specifically to individual country situations, whereas the e-learning courses are broader. 72% noted a change in attitude towards children’s palliative care (scoring 4 or 5 on a scale of 1–5), 73.9% saying their knowledge had increased, 72.5% saying their skills had improved and 61% saying their clinical practice had changed since undertaking the course. Further work on the impact of these courses is ongoing.

Similarly, initial evaluation of the VUCCnet/*e*cancer modules at the point of completion has shown that 99% of participants feel that the information will help improve their professional performance. 71% reported that they found the course easy to access and navigate, and 76% found that the course design and delivery made the subject matter interesting. In view of varying levels of internet provision and the importance of the topic, a text version was also available which to date, has been requested by 15% of learners [41].

## Palliative care education in Africa—the future

The recent WHO 67th World Health Assembly recognised three levels of palliative care education: a basic level aimed at generalists including health-care workers (non-palliative care specialists), an intermediate level and a specialist level. The resolution also focuses on integration of palliative care and highlights the need to include palliative care as an integral part of education and training across the spectrum of care [51].

With the courses outlined within this article, a significant number of health-care professionals across the continent have developed and continue to develop skills and knowledge, enabling a cascade of education potential. Developing palliative care material for Africa should focus on core content for health-care providers such as nurses, doctors, pharmacists, social workers and allied health professionals such as physiotherapists, occupational therapists, lawyers and others as well as general training materials for lay people, policy makers and other stakeholders. The core content for the health professionals should focus on both pre-service and in-service aspects and should include palliative care for both children and adults.

It is possible to combine resources globally to produce effective educational initiatives; creating educational material for a target audience or setting needs close collaboration with all potential stakeholders in order to achieve optimal understanding of the context within which services are delivered in order to achieve maximal effect. Appropriate and responsible use of resources would suggest that such material does not duplicate that already available but, where possible, complements material already present.

Using developing technology to develop education enables creativity and opportunity. The delivery of training needs to utilise the existing e-platforms including those based on the mobile phone technology given the wide coverage of mobile phones in Africa. More resources for palliative care within the region are being developed, and it is important that individuals and organisations collaborate to strengthen resources available, and develop resources in different languages, for example the Spanish materials currently being developed by ‘*Paliativos Sin Fronteras*’ for children’s palliative care.

Clinical placements are an important component of any palliative care training for service providers to put the palliative care approach in practical context. Expansion of opportunity is recognised as palliative care education cascades, however mentorship and support supervision as well as monitoring and evaluating the impact of education and training on patients and families remain key priorities in the region.

There are, however, still various challenges to palliative care education and training in Africa. There are limited clinical sites where health-care providers can obtain clinical experience in delivering palliative care services. It is also important to note that some specialist courses, such as the one which enables nurses and clinical officials to become legal prescribers, are only offered in Uganda, and technical assistance for their adaptation in Africa countries which are considering task shifting is a priority. Mentorship and support supervision of trained providers of palliative care has not been standardised as yet and although a monitoring and evaluation framework for palliative care education and training has been developed, its implementation is yet to be scaled-up across the region.

Combining validated patient outcome measures with evaluation of education programmes is being developed as a way to demonstrate the impact of education for the patient. Competing health priorities in Africa have led to the increasing importance of collecting patient reported outcomes data to demonstrate effectiveness of palliative care and enhance patient centred care models [52–53]. Several outcome measures have been, or are being, validated for this purpose which may be incorporated into educational evaluations in the future. For example, the APCA African POS (Palliative care Outcome Scale) [52–53], the Functional Assessment of Chronic Illness Therapy – palliative care (FACIT-Pal) Scale [54], the Missoula Vitas quality of life index for use among the terminally ill [55] and the Child version of the APCA African POS [56]. African research to underpin African service development and delivery remains a key guiding principle [17, 57].

APCA can report more recent key developments in Africa: the African Union’s ‘Common Position on Controlled Substances and Access to Pain Management Drugs and the Declaration on Non-communicable Diseases (NCDs)’ which includes palliative care [58] and the development of a consensus statement for palliative care integration into health systems in Africa ‘Palliative care for Africa’ [59]. This followed the first African Ministers of Health session in Johannesburg in September 2013 hosted by the South African Deputy Minister of Health, Dr Gwen Ramogkopa [60].

## Conclusion

Despite the challenges, the provision of palliative care education within the region is expanding. With the acceptance of palliative care as an essential component of health care through the WHA resolution, there is an opportunity for increasing education for palliative care across the region, particularly in light of the still increasing burden of cancer. Varying modes of training delivery need to be explored and utilised in order to increase accessibility and availability of training, and through collaboration, scale up can be achieved. However, the impact of training on patient care needs to be evaluated as ultimately, the final point of any education programme is the patient, and any education resource must have improved patient care at its heart [13].

**Table:** Table of organisations and abbreviations used:

APCA	African Palliative Care Association	www.africanpalliativecare.org
APCRN	African palliative care research network	www.africanpalliatvecare.org
HOSPAZ	Hospice and palliative care association of Zimbabwe	www.hospaz.co.zw
HPCA	Hospice and palliative care association of South Africa	www.hospicepalliativecaresa.co.za
IAHPC	International association of hospice and palliative care	www.hospicecare.com
ICPCN	International children’s palliative care network	www.icpcn.org
KEHPCA	Kenya Hospices and Palliative Care Association	www.kehpca.org
MPCU	Makarere palliative care unit	
PACAM	Palliative care Association of Malawi	www.palliativecareassociationofmalawi.org
PCAU	Palliative Care Association of Uganda	www.pcauganda.org
THET	Tropical Health and Education Trust	www.thet.org
VUCCnet	Virtual University for Cancer control	www.cancer.iaea.org/vuccnet
WHO	World Health Organisation	www.who.int
WHPCA	Worldwide Hospice Palliative Care Alliance	www.thewpca.org

## Figures and Tables

**Table 1.** Key supporting documents provided by APCA.

Palliative care core curriculum	Countries and institutions are able to use these documents to integrate palliative care into existing training or in the adaptation or in the adaptation of country-specific national palliative care training packages. APCA is developing generic training materials based on this curriculum and competencies for adaptation and use by many more countries in Africa.
Framework for core competencies	
Beating pain: a packet guide for pain management in Africa [32]	Pain management resources to impact on pain and quality of life.
A handbook of palliative care [33]	
Using opioids to manage pain: a pocket guide for health professionals in Africa [34]	
Standards for provision of quality palliative care across Africa [35]	
Guide to effective teaching methodologies [in press]	
Monitoring and evaluating palliative care education [in press]	To assess the impact of palliative care education.
Two monographs focusing on women with HIV and cervical cancer [36]	Provide a training tool for lay people to fully understand the role of palliative care in HIV and cancer care.

## Online resources

APCA: www.africanpalliativecare.org

Cardiff University palliative care education: http://courses.cardiff.ac.uk/postgraduate/course/detail/p233.html

ICPCN: www.elearnicpcn.org—paediatric palliative care education modules

VUCCnet Palliative care in cancer modules available at: www.ecancer.org/education

WHPCA: www.thewhpca.org (the palliative care toolkit is available here).
